# Efficacy of Integrated Yoga Therapy in Enhancing Cognitive and Cardiac Function in Breast Cancer Patients Undergoing Chemotherapy: A Randomized Controlled Trial

**DOI:** 10.1002/alz70858_097181

**Published:** 2025-12-24

**Authors:** Inbaraj Ganagarajan, Raghavendra M Rao, Kaviraja Udupa, Amritanshu Ram, Jamuna G Rajan, Sathyaprabha T N

**Affiliations:** ^1^ National Institute of Mental Health and Neuro Sciences (NIMHANS), Bengaluru, Karnataka, India; ^2^ Central Council for Research in Yoga and Naturopathy, New Delhi, Delhi, India; ^3^ HealthCare Global Cancer Hospital, Bengaluru, Karnataka, India

## Abstract

**Background:**

Cognitive impairment and cardiac dysfunction are common adverse effects of chemotherapy in breast cancer (BC) patients, with no established interventions to address these issues. Yoga, known for its benefits on cognitive and cardiac health, may offer a complementary approach. This study examines the effects of an 18‐week yoga regimen on cognition and cardiac function in BC patients undergoing chemotherapy.

**Method:**

A total of 150 BC patients scheduled for chemotherapy were randomized into a Treatment as Usual group (TAU) (*n* = 75) or Treatment as Usual with Yoga group (TAUY) (*n* = 75). The 18 week integrated yoga regimen included asanas, pranayama, meditation, and relaxation. Assessments were conducted pre‐chemotherapy and after six cycles of chemotherapy, focussing on: a) cognition using neuropsychological test batteries to assess various domains of cognition, including learning, memory, recall, focused attention, motor speed, planning, and executive functioning; b) serum brain‐derived neurotrophic factor (BDNF); c) serum cortisol; d) heart rate variability (HRV); e) echocardiography; f) perceived stress; g) Quality of Life assessments (FACT COG & EORTC QLQ C30).

**Result:**

At baseline, both groups exhibited mild cognitive changes, moderate stress and, altered cardiac autonomic functioning. After six chemotherapy cycles, the control group experienced significant cognitive decline across all domains and reduced serum BDNF levels. They also exhibited severe stress, elevated cortisol, and reduced HRV and quality of life. Conversely, the yoga group maintained cognitive capabilities, with notable improvements in attention, working memory, and mental speed. BDNF levels were significantly higher in the yoga group, correlating positively with cognitive performance. Emotional function (EORTC) was inversely correlated with LF/HF ratio, indicating enhanced autonomic balance in the yoga group. Additionally, perceived stress strongly correlated with serum cortisol, suggesting that stress reduction through yoga contributed to improved cognitive resilience.

**Conclusion:**

Yoga helps alleviate chemotherapy‐induced cognitive and cardiac issues, promoting cognitive preservation and cardiac balance in BC patients undergoing chemotherapy. Further studies are required to validate these findings, explore underlying mechanisms, and optimize the yoga approach.

